# A comprehensive review on traditional uses, phytochemistry and pharmacological properties of *Paeonia* *emodi* Wall. ex Royle: current landscape and future perspectives

**DOI:** 10.1186/s13020-023-00727-7

**Published:** 2023-03-02

**Authors:** Nida Zahra, Javed Iqbal, Muhammad Arif, Banzeer Ahsan Abbasi, Hassan Sher, Ayesha Fazal Nawaz, Tabassum Yaseen, Alibek Ydyrys, Javad Sharifi-Rad, Daniela Calina

**Affiliations:** 1 Department of Biotechnology, University of Mianwali, Mianwali, 42200 Pakistan; 2 Department of Botany, Bacha Khan University, Charsadda, Khyber Pakhtunkhwa Pakistan; 3 Department of Botany, Rawalpindi Women University, 6th Road, Satellite Town, Rawalpindi, 46300 Pakistan; 4 Center for Plant Sciences and Biodiversity, University of Swat, Kanju, 19201 Pakistan; 5 National Institute of Genomics and Advanced Biotechnology (NIGAB), National Agricultural Research Center (NARC), Park Road, Islamabad, Pakistan; 6 Biomedical Research Centre, Al-Farabi Kazakh National University, Al-Farabi Ave. 71, 050040 Almaty, Kazakhstan; 7 The Elliott School of International Affairs, George Washington University, 1957 E St NW, Washington, DC 20052 USA; 8 Facultad de Medicina, Universidad del Azuay, Cuenca, Ecuador; 9 Department of Clinical Pharmacy, University of Medicine and Pharmacy of Craiova, 200349 Craiova, Romania

**Keywords:** *Paeoniaemodi*, Traditional uses, Phytochemistry, Pharmacological activities

## Abstract

*Paeonia* *emodi* Wall. ex Royle is commonly known as Himalayan *paeony* has great importance as a food and medicine. The practice of *Paeonia* *emodi* Wall. ex Royle is very ancient and it is conventionally used for a wide range of illnesses in the folk system of medicine because of its wide beneficial phytochemical profile. The main purpose of the current review was the synthesis of recent data on botany, ethnopharmacology, phytochemistry and potential pharmacological mechanisms of action of *Paeonia* *emodi* Wall. ex Royle, thus offering new prospects for the development of new adjuvant natural therapies. Using scientific databases such as PubMed/MedLine, Scopus, Web of Science, ScienceDirect, Google Scholar, Springer, and Wiley, a comprehensive literature search was performed for *Paeonia* *emodi* Wall. ex Royle. For searching, we used the next MeSH terms: “Biological Product/isolation and purification”, “Biological Products/pharmacology”, “Drug Discovery/methods”, “Ethnopharmacology, Medicine”, “Traditional/methods”, “Paeonia/chemistry”, “Plant Extracts/pharmacology”, “Phytochemicals/chemistry”, “Phytochemicals/pharmacology”, “Plants, Medicinal”. The results of the most recent studies were analyzed and the most important data were summarized in tables and figures. Phytochemical research of *Paeonia* *emodi* Wall. ex Royle has led to the isolation of triterpenes, monoterpenes, phenolic acids, fatty acids, organic compounds, steroids, free radicals and some other classes of primary metabolites. In addition, diverse pharmacological activities like antibacterial, antifungal, anticoagulant, airway relaxant lipoxygenase and beta-glucuronidase inhibiting activity, radical scavenging activity, phytotoxic and insecticidal activities have been reported for *Paeonia* *emodi* Wall. ex Royle. Different bioactive compounds of *Paeonia* *emodi* Wall. ex Royle has proven their therapeutic potential in modern pharmacological and biomedical research to cure numerous gastrointestinal and nervous disorders. In future, further in vitro and in vivo therapeutic studies are required to identify new mechanisms of action, pharmacokinetics studies, and new pharmaceutical formulations for target transport and possible interaction with allopathic drugs. Also, new research regarding quality evaluation, toxicity and safety data in humans is needed.

## Introduction

*Paeonia* *emodi* commonly called “Himalayan Peony*”* is found in the northern territories of Pakistan [1, 2]. *Paeonia* *emodi* is a medicinal plant that belongs to the Paeoniaceae family [3]. It is renowned as "the queen of herbs" [4]. *Paeonia* *emodi* Wall. ex Royle is a versatile medicinal plant of major relevance in the Himalayan region [5]. The plant is widely distributed in Pakistan, India, and Afghanistan. *Paeonia* was named after a Greek legend about a medical student named Paeon who healed Pluto's wound. Pluto later spared *Paeon* from death by transforming him into the peony, a medicinal plant that is still used today [6]. In different regions, different vernacular names are used for *Paeonia* *emodi* Wall. ex Royle for example in Urdu it is undsalib, in English it is called Paeoney rose, Himalayan paeony and in hindi it is called Pawin, Chandayra, Ud-salap [4, 6]. Because of its high therapeutic worth, the plant is intensely gathered in the region. Monoterpene glycosides, lactiflorin, paeoniflorin, peoninol, oxypaeflorine are some compounds that are reported from this plant. Previously monoterpene glycosides have been reported from the roots of *Paeonia* *emodi* Wall. ex Royle [7, 8]. Oleanolic acid, betulinic, ethyl gallate, methyl grevillate, and 1,5-dihydroxy-3-methylanthraquinone are some of the components extracted from this plant. [9]. Different constituents are monoterpene glycosides, wurdin and 15 benzoyl wurdin alongside paeoniflorin, lactiflorin and oxypaeoniflorin [7]. The different other constituents extracted from this plant incorporate a β-glucuronidase-repressing triterpene, Pa 11,beta,5alpha,23,24-pentahydroxy-30-norolean-12,20(29)-d ien-28-oic acid, oleanolic acid, betulinic acid, ethyl gallate, methyl grevillate and 1,5-dihydroxy-3-methylanthraquinone [9]. Different constituents are monoterpene glycosides, wording and benzoylwurdin alongside paeoniflorin, lactiflorin and oxypaeoniflorin [7].

The current review is an updated and novel report of traditional uses, pharmacology, the potential mechanism of actions and phytochemical constituents of *Paeonia* *emodi* Wall. ex Royle*.* This review paper provides a recent comprehensive literature review on the importance of its conservation and future economical sustainability. It additionally features the logical reason for future research on *Paeonia* *emodi* Wall. ex Royle and its genuine potential for the improvement of the market for homegrown therapeutic items.

## Methodology

The relevant literature was collected through a bibliographic investigation conducted in the next scientific databases PubMed/MedLine, Scopus, Web of Science, ScienceDirect, Google Scholar, Springer, and Wiley using the next MeSH terms: “Biological Product/isolation & purification”, “Biological Products/pharmacology”, “Drug Discovery/methods”, “Ethnopharmacology, Medicine”, “Traditional/methods”, “Paeonia/chemistry”, “Plant Extracts/pharmacology”, “Phytochemicals/chemistry”, “Phytochemicals/pharmacology”, “Plants, Medicinal”.

Inclusion criteria: (i) relevant papers which included traditional uses, phytochemistry and modern pharmacological studies (ii) studies who included in vitro and in vivo experiments along with potential mechanisms of action (iii) papers written in the English language.

Exclusion criteria: (i) duplicates and incomplete information, ii) abstracts, letter to the editor, short communications, (iii) experiments made using homeopathic preparations associated, (iv) studies written in another language than English. Chemical constituents of the plant were identified, IUPAC names and structural and chemical formulas were confirmed from ChemSpider and PubChem. The taxonomy of the plant has been validated according to WFO [10].

### Botany

**Fig. 1:**
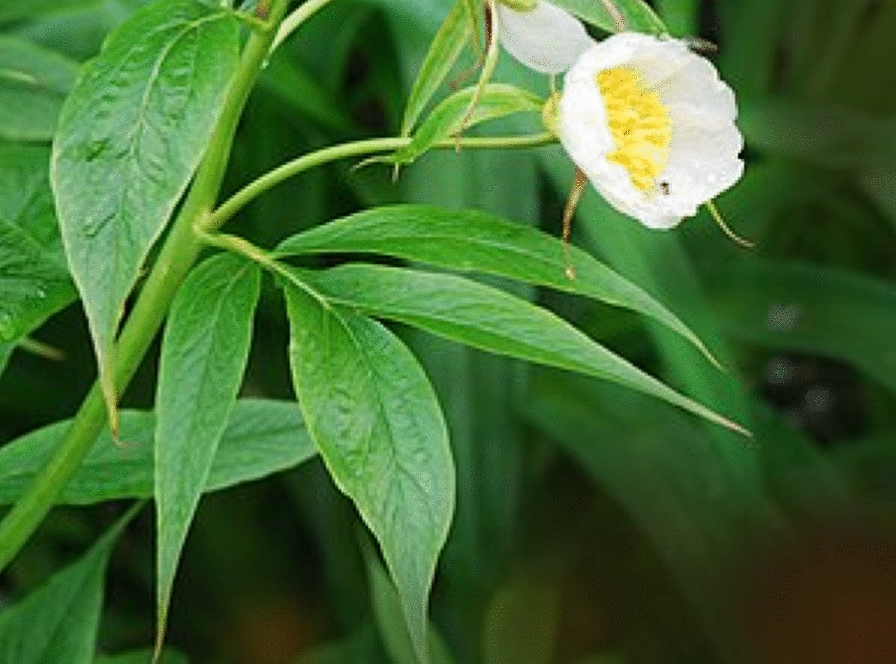
The medicinal plant *Paeonia* *emodi*

*Paeonia* *emodi* Wall. ex Royle. has ternary or bi-ternary, glabrous leaves; blossom singular, white, or pale pink flowers [11]. It's a glabrous perennial herb with thick tuberous roots that grow in clumps and leaves are arranged in a ternate pattern, with decurrent whole or incised leaflets. Flowers are white-coloured (25–10 cm across) and have black, silky, and gleaming seeds [1]. As defined its botany differently is enduring herbs, up to 70 cm tall [12]. The stem is smooth. Proximate leaves 2-ternary; a few handouts fragmented; pamphlets and sections up to 15, oval elliptic or ovoid lanceolate, 9–13 × 2–3.5 cm, the two surfaces smooth, base cuneate, decurrent, highest point hone. Blooms 2–4 for each shoot, both terminal and axillary, single, 8–12 cm wide, all or simply terminal one made. Bracts 3–6, leaf-like, lanceolate. Sepal's ca. 3, suborbicular, ca. 1.5 × 1.5 cm, zenith caudate. Petals white, obovate, ca. 4.5 × 2.4 cm. Filaments 1.5–2 cm. Plate a nular. Carpel 1(or 2), light yellow tomentose, now and again glabrous. Follicles ovoid, 2–3.5 × 1–2 cm. Seeds dull, globose. The blooming period is from May to March. Leaves are ternary with lamina pale. Blooms are particular, axillary and of red concealing. Bracts are verdant, petals are 8 and seeds move from 3–5 [13]. It bears oblong-lanceolate leaves that are glabrous on both sides [14]. Under a microscope, the foliar epidermis is made up of irregularly shaped epidermal cells with undulating walls. Adaxial epidermal cells measure 71.5 m in length and 73.5 m in breadth, whereas abaxial epidermal cells measure 88.5 m in length and 76 m in width. Stomata are often anomocytic, with varying lengths and widths [15]. Flowers are white and are arranged in a terminal or axillary arrangement on branches. The blooms are mostly bracteates, with suborbicular sepals and obovate petals. The fruit is a follicle that contains ovoid seeds that are lobose black. Pollens are tricolporate, monad, and spherical in polar viewpoint, but perprolate in equatorial perspective (Fig. 1). The pole diameter (polar view) is 38.14 m, the equatorial diameter is 30.87 m, the P/E ratio is 1.23 m, the colpi length is 12.3 m, the width is 15.83 m, and the exine thickness is 2 [4].

It propagates healthy in high altitude cool, climate zones; displays well in deep, loamy, and humid soil. The plant can be found growing in narrow valleys or glens with streams. To thrive, the plant requires a lot of moisture and nutrients. The plant flourishes in the shade of *Juglans* *regia* (walnut) and *Populus* *deloides* (cottonwood poplar). *Paeonia* is a food source for the locals. After being collected from the forest, the leaves are cooked and kept in the shape of leaf cakes for a long time. Paeonia is eaten raw, as well as fermented and sun-dried. It is an ancient, indigenous treatment as well as a traditional technique utilized by the Bhotiya tribal community to treat stomach issues.

### Ethnopharmacology

In the current period of science and innovation, individuals in the creating nations depend on conventional arrangements of medicinal services because of two main reasons, i-e low price and fewer symptoms contrasted with cutting-edge allopathic medications [16–18]. For the treatment of different sicknesses, plants have been used from the beginning of time. In the developing world, traditional practices are an important part of the primary healthcare system [19, 20].

*Paeonia* *emodi* Wall. ex Royle has been used for therapeutic purposes since ancient times. Today, most of the ethnopharmacological data of the *Paeonia* *emodi* Wall. ex Royle species is found in traditional Chinese medicine [6]. The Chinese used *Paeonia* *emodi* Wall. ex Royle to treat inflammation and hypertension. The Chinese boiled the root of the white peony and used the obtained decoction in various ailments such as joint pain, hepatitis, muscle cramps, nervous disorders, cardiovascular diseases, gynecological ailments. Its uses are recorded in the ancient Chinese medicine book "Eastern Han Dynasty" from AD 25-220 [21]. Information about this plant also appears in the "Pharmacopea of the People's Republic of China" [22]. Anti-inflammatory, analgesic, immunomodulatory, antioxidant, sedative and antimicrobial properties were attributed to the *Paeonia* *emodi* Wall. ex Royle [23].

In the local and old-fashioned systems of medicine, *Paeonia* *emodi* Wall. ex Royle has extensively been used because of its wide beneficial profile. *Paeonia* *emodi* Wall. ex Royle has been broadly used in traditional treatment for a broad range of illnesses like Stomach problems, muscle problems, intestine problems, fever, pain killers and headaches. The plant is also traditionally used for uterine illnesses, colic, epilepsy, convulsions, hysteria, obstructions, and dropsy. The whole plant is highly medicinal as a mixture of dried flowers is extremely helpful in diarrhea while seeds are cathartic and emetic [24]. Its roots, stem, leaves, seeds and flowers are used medicinally in different forms and sometimes in combination with other herbs. The tubers of *Paeonia* *emodi* Wall. ex Royle is valuable for uterine ailments, colic, nauseous impediments, dropsy, epilepsy, seizures and madness and is likewise given to kids as a blood purifier [25]. The underground plant parts are utilized to fix spinal pain, edema and brain abnormality and are likewise utilized as an energizer, vomit inducer, cleansing, blood purifier and bellyache while seeds are laxative [26]. It is likewise utilized in spine hurt, tonic, emetic, cleansing, and blood purifier. It is additionally utilized in dropsy, epilepsy and colic and body tonic.

Detailed information about plant parts used, ailments treated and mode of utilization are provided in Table 1.

**Table 1:** Different ailments treated by *Paeonia* *emodi* Wall. ex Royle and their mode of utilization

Country	Local name	Part used	Ailments	Mode of utilization	Refs.
Pakistan	–	Rhizome	Backache and tonic	–	[27]
	Mamekh Ud-e-Saleeb	Roots, tuber, flowers, Seeds, fruit	Stomach problems, muscles problems, intestine problems, fever, pain killer, headache	–	[28]
	Mamakhi	Leaves	Epilepsy, blood purifier, indigestion, headache, dizziness, vomiting	Boiled extract	[29]
		Flowers	General body tonic and diarrhea	Decoction	[12]
		Rhizome	Backbone ache, tonic, cathartic		[11]
			Blood purifier, epilepsy, colic, blood tonic		
			Back pain and common weakness		[30]
	Himalayan peony	Flower	Antidiarrheal, hemorrhoids, expectorant, antispasmodic		[31]
	Mamekh	Seeds, tuber	Rheumatism and backache	Paste and powder	[29]
		Rhizome, seeds	Backache, general weakness, blood purifier, tonic	Decoction	[32]
			Epilepsy, cathartic, colic, purgative		
		Roots	Internal injuries	Paste	[33]
			Diarrhea, rheumatic pain, gynecological disorders, vomiting	Powdered	[34, 35]
		Roots, rhizome	Backbone ache, tonic, cathartic, epilepsy, purify blood	Paste, extract decoction	[36]
		Rhizome	Stomach problems	Extract	[37]
	Mamekh	Tuber, flowers, seeds, petals	Hysteria, uterine diseases, colic, convulsions, bile duct problems hemorrhoids, varicose veins problem, hypertension, obstruction, blood purifier, cathartic, diarrhea, cough	Infusion and decoction	[35] [38]
		Whole plant –	Dysentery	Decoction	[39]
			Tonic/analgesic		[26]
		Tuber, seeds, flower, whole plant	Nervous diseases, uterine diseases, colic, bilious obstruction, dropsy, epilepsy, convulsions, hysteria, diarrhea vomiting, cholera, eye diseases, tuberculosis		[35] [38]
		Stem, tuber	Joint pain, bone fractures, epilepsy, convulsions, hysteria, colic, uterine diseases, bilious obstructions, dropsy, blood purifier	Powder and paste	[40] [38]
		Tuber, flowers	Epileptic attacks, cholera, whooping, cough, uterus diseases, colic, bilious, obstruction, dropsy, convulsions, hysteria, diarrhea, cathartic	Infusion	[35]
		Rhizome	Backache, general weakness, skeletomuscular problems	Powder	[41]
			Wounds, cuts, narcotic, tonic, tumor, anticancer, stimulant		
	Chandra	Roots, shoots, leaves	Whooping, cough, intestinal spasms, cuts, post-natal care	Infusion and paste	[42]
	Mameikh	Tuber, flowers, seeds, roots, twigs, leaves	Nervous disorders, stomach complaints, purgative	Juice	[43]
			Body pain, uterus disorder, blood purifier, skin diseases, backache weakness		[44] [38]
	Mamaikh	Rhizome, seeds	Backbone ache, dropsy, epilepsy, cathartic, blood purifier, colic, purgative, tonic	Extract	[45] [35]
		Rhizome	Backbone ache, tonic, cathartic, blood purifier, dropsy, epilepsy, colic		[46] [35]
	Pamekh, Mamekh	Rhizome	Anti-rheumatic, stomach ailments	Extract	[47]
		Rhizomes, roots and seeds	Backbone ache, dropsy, epilepsy, tonic, cathartic, blood purifier		[48]
		Rhizome and roots	Purgative, headache, dizziness, vomiting, pregnancy, cathartic backache, headache, dizziness, vomiting, edema, epilepsy, therapeutic, blood cleanser, helps in pregnancy laxative, bellyache		[49]
	Mamekh	Rhizome	Backache and stimulant	–	[27]
		Shoot	Body pains, heals fractured bones	–	[50]
China	–	Stem, tuber	Joint pain, bone fractures, epilepsy, convulsions, hysteria, colic	Powder and paste	[40] [35]
Ethiopia	Chandra	Leaves	Uterine diseases, bilious obstructions, dropsy, blood purifier	–	[51]
India	Chandra	Tuber, flowers	Epileptic attacks, cholera, whooping, cough, uterus diseases	Infusion	[24] [38]
			Indigestion, seizures, dropsy, epilepsy, mania		
			Mental disorder, rheumatism, urine complaints		
		Leaves	Dysentery, blood purifier		[1]
	Dhandaru	Roots	Skin diseases	Paste	[52]
	Chandra	Roots	Intestinal pain, dysentery, piles	Decoction	[53]
			Epilepsy, cathartic, colic, purgative		
		Tuber and roots	Bilious, obstructions, biliousness	-	[54]
		Leaves, roots	Blood dysentery, diabetes, improve lactation, hysteria, epilepsy	Powder	[55] [38]
		Roots, rhizome	Uterine diseases, biliousness, dropsy, nervous system	Infusion	[56]
			Blood purifier, cathartic, diarrhea, cardiovascular		
			Headache, hysteria, abdominal spasms, nervine tonic		
			Respiratory illnesses, high blood pressure, atherosclerosis		
	Bhoi, Pawin	Roots	Stomach problems	Decoction	[57]
	Udsaleeb	Roots, stem, leaves	Dyspepsia, dysentery, diarrhea, fever, blood, purifier	–	[58] [38]
			Rheumatism, urinary troubles, colic, convulsions		
			Dropsy, cuts, ulcers, wounds, mental diseases		
	Chandra	Leaves	Blood purifier, dysentery, digestive disorders, foul ulcer	Boiled fried	[59]
			Leucorrhoea	Decoction	[55]
		Leaves and root	Epilepsy	Powder	[60]
		Leaves	Dysentery, haemoglobin deficiency	–	[59] [38]

#### Frequently treated ailments

**Fig. 2:**
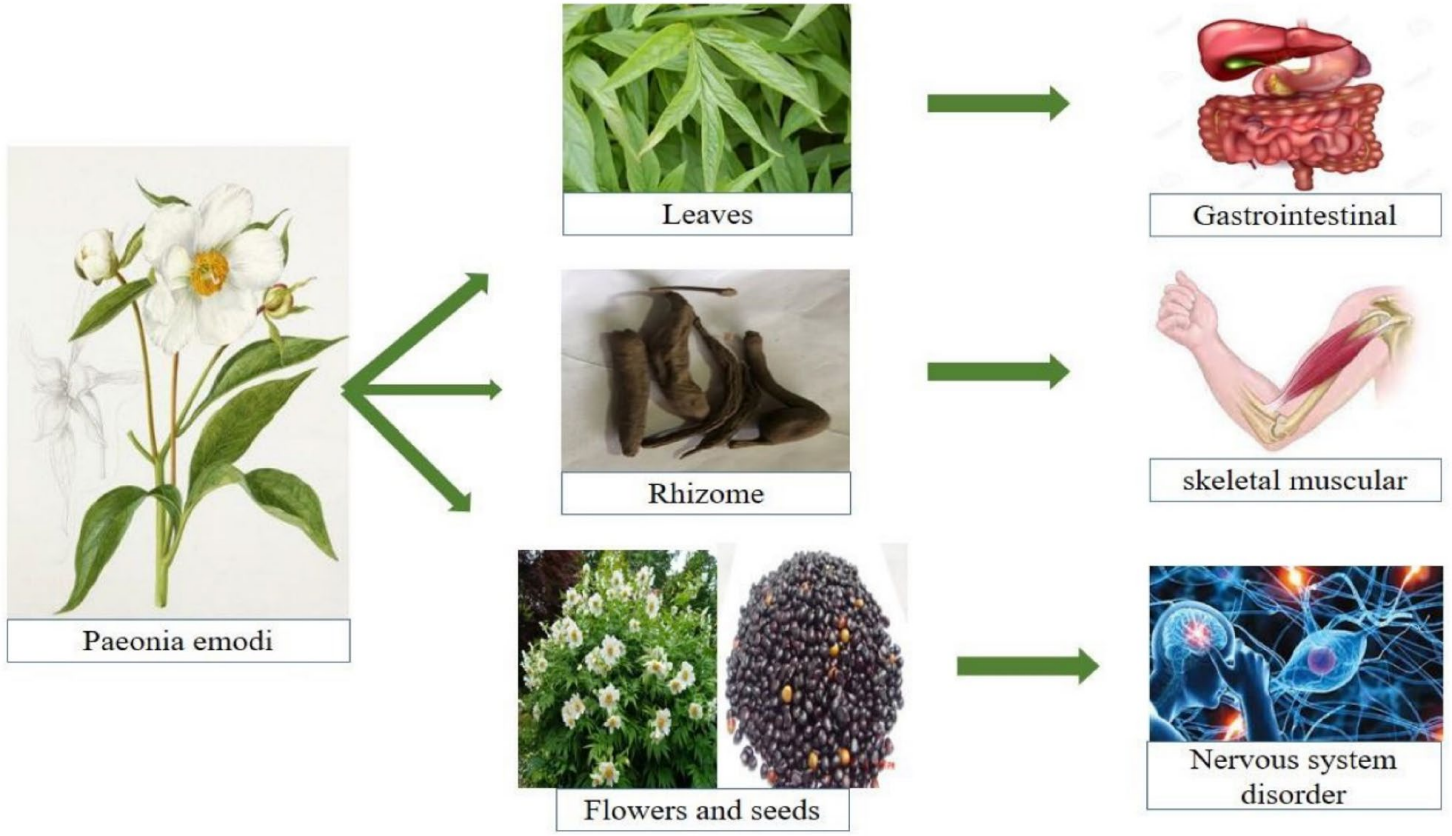
Major aliments treated by *Paeonia* *emodi* Wall. ex Royle

Among frequently treated ailments, the top-listed category was treated by *Paeonia* *emodi* Wall. ex Royle extracts, were gastrointestinal diseases, followed by skeletomuscular and nervous disorders; other common diseases include fever, headache, wounds, renal/urinary, gynecological, bone disorders, psychiatric disorders, respiratory and liver complaints, cardiovascular and cancer. Numerous mediators have provided detail about the pharmacokinetic importance of plants used in gastrointestinal messes [61–72]. The leaves and roots of *Paeonia* *emodi* Wall. ex Royle is used to treating many GIT problems in the form of decoction [1, 51, 57]. *Paeonia* *emodi* Wall. ex Royle rhizome powder is also used to treat pain [73]. Because of the accessibility of plants with dynamic fixings, basic oil and mixes that are profoundly compelling against colitis, gastritis, intestinal worms and disease, and so on, as industrious by ethnopharmacological considers, plant species for the cure of gastrointestinal problems play an important role in traditional medicines in the Madonie Mountains. [74–77]. Figure 2 shows major ailments treated by *Paeonia* *emodi* Wall. ex Royle.

#### Plant parts used

**Fig. 3:**
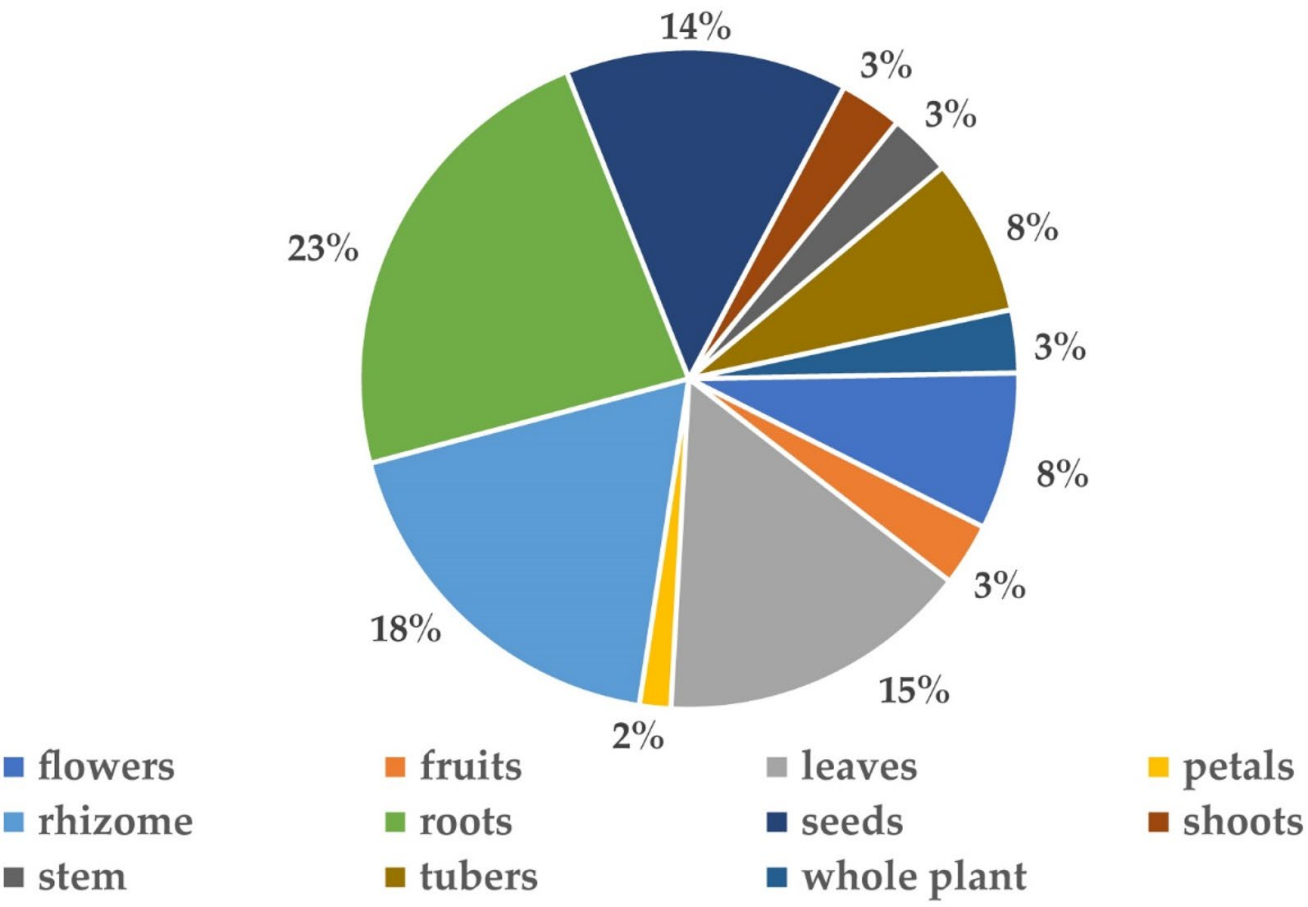
Different plant parts used in *Paeonia* *emodi* Wall. ex Royle

Most of the time entire plant and in many circumstances various plant parts, including root, stem, leaves, rhizome, bark, tuber, and seeds are used for the cure of different ailments. In many studies, various parts of the plant are blended for making readiness which is then used as a drug. Roots are the most often used plant parts (23%), followed by flowers (18%), leaves (15%), rhizomes (14%), tubers (8%), fruits, stem, shoots (3%) (Fig. 3). Rhizome was the main medicinal part that is used for the treatment of different diseases. For the preparation of medicine, several parts of the individual plant were used, among all 38% of species were used for their rhizome/roots [53]. Rhizome was generally used for the treatment of backache [27, 30]. Roots of *Paeonia* *emodi* Wall. ex Royle was used for treating many skin diseases [52]. The same results were obtained in the present review that rhizomes and roots were the most frequently used part for the preparation of different medicines to cure many ailments.

#### Preparation and administration

**Fig. 4:**
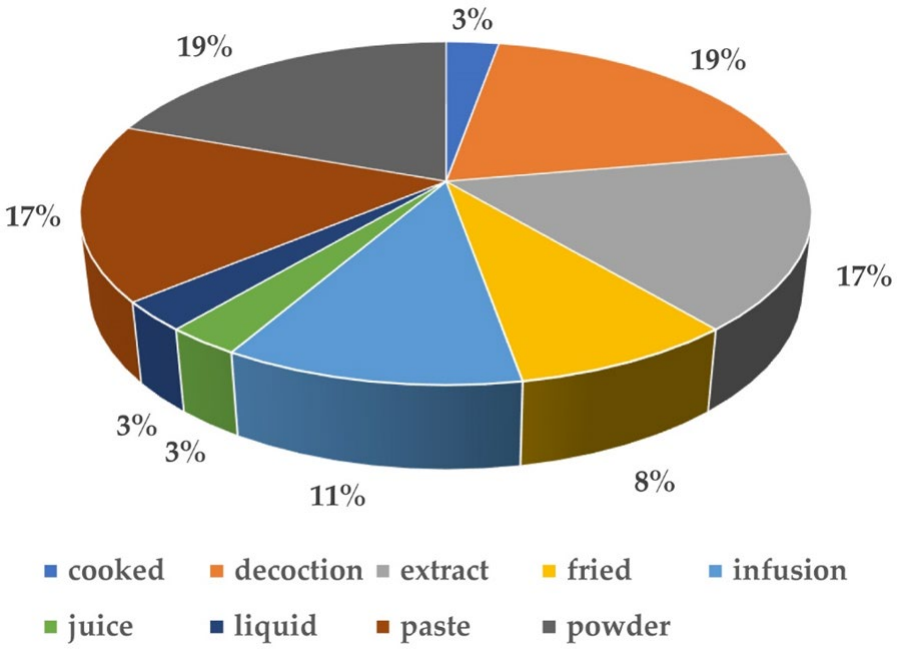
Preparation and mode of administration

For the administration of herbal medicinal plants, distinctive readiness strategies are utilized which include decoction, combination, powder, squeeze, and glue. In our study main method of preparation is decoction, powder (19%), paste, extract (17%), followed by infusion (11%), fried (8%), cooked (3%), and juice (3%) (Fig. 4). The most widely recognized strategy for arrangement is decoction and mixture which reveals comparative discoveries [78–80]. A decoction is the major primary prescription form in Ayurveda [81]. It is created by boiling or heating the necessary plant material in water, whereas distillation is created by hanging the necessary plant material in either warm or cold water. After the plant has completely dried, the powder is produced by crushing the entire plant or a part of it. Huge numbers of arrangements are made by utilizing water as a dissolvable medium. Many people utilize castor oil, coconut oil, ginger, neem, and mustard oil in the creation of adhesive and ointment. The most common way of taking medicine is oral ingestion for internal use but in some ailments, topical application is frequently employed in the form of paste. Decoction of the leaves is administered for dyspepsia, jaundice, and cardiac and respiratory problems and is used as a tonic [82]. The major mode of utilization decoction and infusion is frequently taken as tea and broths [79, 83]. The decoction of the flowers is given for diarrhea and used as a general body tonic while root decoction is administered for various stomach problems [12, 57]. The nutritional value of *Paeonia* species flowers is well known. *Peony* blooms are high in protein, sugar, superoxide dismutase, and other nutrients, according to studies. [6]. Our results of the current review matched with the results of previous reviews in the way that the most frequently used part of *Paeonia* *emodi* Wall. ex Royle is rhizome and roots and the major mode of utilization is decoction. It might be because the rhizome is hard and its decoction is a preferred use. In previous literature of ethnomedicinal studies, the decoction was frequently used due to the activation of some chemical compounds upon heating.

For treatments of diverse diseases, the use of plant extract and its associated phytochemicals are of great importance [20, 84, 85]. Worldwide different studies have been conducted to confirm the pharmacological and medicinal effects of these phytochemicals [86–88] As per the World Health Organization, the best source to get an assortment of medications was therapeutic plants. Therefore, investigations were conducted to understand the properties, efficacy, and safety [89, 90]. The investigation of organically dynamic mixes from plants had consistently been of incredible interest to researchers [91–93]. Several constituents were isolated from *Paeonia* *emodi* Wall. ex Royle which shows significant sedative and anti-inflammatory activities [94]. The ethanolic concentrate of *Paeonia* *emodi* Wall. ex Royle was fractioned into n-hexane, ethyl acetic acid derivation, and dichloromethane while its aqueous fraction was left for phytochemical screening [2]. From the tuber of *Paeonia* *emodi* Wall. ex Royle large numbers of secondary metabolites were isolated which were used for the treatment of numerous diseases like rheumatism, uterine diseases, nervous disorders etc. [2, 94]. Qualitative phytochemical screening of *Paeonia* *emodi* Wall. ex Royle rhizome contains a large number of compounds such as carbohydrates, phenolics, tannins, reducing sugars, cardiac glycosides, anthraquinone glycosides, terpenes, steroids, resins and oxalic, tartaric, citric and ascorbic acids [95]. In *Paeonia* *emodi* Wall. ex Royle the absolute phenolic substance of hydroalcoholic extricate was seen as 375.83 ± 3.82 (mg GAE/g) while that of aqueous extract was 187.83 ± 2.52 (mg GAE/g) [95]. Previously from the roots of *Paeonia* *emodi* Wall. ex Royle monoterpene glycosides and triterpene were reported [94].

#### Triterpenes

In the previously conducted studies, different triterpenes were identified. In the present review, it comprises 22.7% out of all investigated phytochemicals. Primary triterpenes include emodinol, β-amyrin, lupeol, 24-methylenecycloartanol, cycloartenol, betulinic acid and oleanolic acid. Monoterpenes glycosides [7] and triterpenes [9] from *Paeonia* *emodi* Wall. ex Royle was reported in other studies. For the triterpenes, Emodinol was the first time confined from the chloroform dissolvable part of *Paeonia* *emodi* Wall. ex Royle and it showed significant β-glucuronidase inhibitory action [96]. Betulinic acid, oleanolic acid are some of the components isolated from this plant [9]. Triterpenes were the principal class of compound occurring in *Paeonia* *emodi* Wall. ex Royle. Some triterpenes were reported from time to time from the extract of this plant e.g. Emodinol [56]. Oleanolic acid, a triterpene, has been demonstrated to have anticoagulant, cardioprotective, calming effect, lipoxygenase and glucuronidase inhibitory, free-radical scavenging activities as well as significant herbicidal and antibacterial activity. 24-methylenecycloartanol was triterpene determined anti-inflammatory activity while the same chemical was reported by [56] similar pharmacological activities. β-amyrin and lupeol are two important triterpenes that showed anti-bacterial activity while the former also showed antiulcer properties [4].

#### Monoterpenes

In the previously conducted research studies, different monoterpenes were identified. In the present review, 12.1% of monoterpenes have been identified from all the phytochemicals. The primary monoterpenes isolated are lactiflorin, paeonin (A, B, C), wurdin, benzoyl wurdin, oxypaeoniflorin, paeoniflorin [94]. Paeonin A and paeonin B were segregated as a dismal sticky solid [96]. From the chloroform-dissolvable portion of *Paeonia* *emodi* Wall. ex Royle roots, Paeonin A and B and some new monoterpenes galactosides were isolated which showed potent lipoxygenase inhibitory activity [96]. Recent research studies on *Paeonia* *emodi* Wall. ex Royle investigated main constituents such as monoterpenes, triterpenes, and polyphenols and showed potential biological activities such as chemopreventive, cytotoxic, and cardioprotective activities [94]. According to [7] the main constituents in the phytochemical of *Paeonia* *emodi* Wall. ex Royle were monoterpenes paeoniflorin, lactiflorin, and oxypaeoniflorin are among the glycosides, wurdin, and benzoylwurdin. Numerous biological effects were ascribed to the specific chemotaxonomic indicators, paeoniflorin, and their derivatives, which were monoterpenes with a pinane skeleton [97]. Wurdin and benzoylwurdin were the two important monoterpenes showing anticoagulant activity, lipoxygenase and glucuronidase inhibitory, and free radical scavenging activities, cardioprotective and relaxing properties [56] while the same monoterpene was reported by [98] showing significant herbicidal and antibacterial activities. The crude extract of *Paeonia* *emodi* Wall. ex Royle showed maximum inhibitory activity against an obligate parasite *S.* *typhi* and a gram-negative bacteria *S.* *flexeneri* while *Paeonia* *emodi* Wall. ex Royle aerial portions had strong herbicidal action but no antifungal or antibacterial activity [48]. The two important monoterpenes (oxypaeoniflorin, Paeoniflorin) showed phytotoxic activity against *Lemna* *aeguinoctailis* and anticoagulant, cardiovascular, lipoxygenase, β-glucuronidase inhibitory, free radical scavenging and antibacterial activities [24, 56, 98]

#### Phenolic acids

Phenolic acid was important phytochemical derived from the aerials parts of *Paeonia* *emodi* Wall. ex Royle. In all previously conducted studies, different types of phenolic acid were identified. The present review divulges 16.6% of total phytochemicals. Paeonol, hydroxybenzoic acid, gallic acid, methyl gallate, ethyl gallate, methylgrevillate, benzoic acid, 3-hydroxy benzoic acid, 4-hydroxy benzoic acid, paeoninol, oligostilbene, and chrysophanic acid are the most common phenolic acids. [8, 56, 98]. From the extract of *Paeonia* *emodi* Wall. ex Royle specific amounts of phenol were determined using the Folin-ciocalteu reagent [99]. Paeonol and hydroxybenzoic acid were two important phenolic acids that showed inhibitory potential against the enzyme lipoxygenase and anti-oxidant activity [8]. According to [56], Gallic acid has anticoagulant, cardiovascular, and relaxing actions, as well as inhibitory and free radical quenching properties for lipoxygenase and β-glucuronidase. While the same chemical exhibit inhibitory potential against the enzyme lipoxygenase and anti-oxidant activity [8]. The phenolic acid and ethyl gallate showed significant herbicidal, antibacterial and anticoagulant activity [98]. Benzoic acid, 3-hydroxy benzoic acid, and 4-hydroxy benzoic acid all have similar anticoagulant, cardioprotective as well as inhibitory and scavenging properties for lipoxygenase, β-glucuronidase, and free radical scavenging properties. [56].

#### Fatty acids

Fatty acid constitutes 13.6% of the present study. Out of all the phytochemicals studied, the primary fatty acids include octanoic acid, decanoic acid, lauric acid, myristic acid, palmitic acid, palmitoleic acid, stearic acid, oleic acid, and linoleic acid [100]. The root oil of *Paeonia* *emodi* Wall. ex Royle was analyzed by [100] in which octanoic, decanoic, lauric, palmitic, stearic, oleic, linoleic, palmitoleic, myristoleic, and myristic acids were found in the saponifiable lipid. The octanoic acid showed significant antifungal and antioxidant activities. Both decanoic and lauric acid exhibit antibacterial activity while the former also exhibits antioxidant activity [100]. Probably all the fatty acids given in the table showed maximum antibacterial and antioxidant activity.

#### Other compounds

Previously, steroids were reported as 3% in plant. Campesterol and sitosterol were reported in the root oil of *Paeonia* *emodi* Wall. ex Royle and determined the antibacterial, antifungal and antioxidant activities [100]. Other organic compound constitutes 7.5% of all the compounds present in *Paeonia* *emodi* Wall. ex Royle*.* The major organic compounds are divergioic acid, chrysophanic acid, benzoylwurdin, and dichloromethane [2, 98, 101]. Divergonic acid was an important organic compound that demonstrated cytotoxicity against disease cell lines [101]. Chrysophanic acid and benzoylwurdin were reported by [98] and both organic compounds exhibited significant herbicidal and antibacterial potentials in *Paeonia* *emodi* Wall. ex Royle*.* Further, [2] reported free radicals scavenging, antioxidant, antibacterial, and antifungal potentials using the organic compound Dichloromethane. The other phytochemicals reported in *Paeonia* *emodi* Wall. ex Royle was d-galactose, baicalein, norhederagenin, DPPH, methyl grevillate and hydrogen peroxide [2, 8, 40, 96, 98, 101] which constitute 7.5% of all phytochemicals investigated. D-galactose was a monosaccharide sugar that showed inhibitory potentials against lipoxygenase and anti-oxidant activity [8]. The flavonoid  baicalein exhibited potent lipoxygenase inhibitory activity in *Paeonia* *emodi* Wall. ex Royle [96]. Norhederagenin, a metabolite showed significant cytotoxicity against malignancy [101]. An antioxidant compound 2,2-diphenylpicrylhydrazyl displayed scavenging antioxidant activity in *Paeonia* *emodi* Wall. ex Royle [40]. Methyl grevillate was an important alkaloid that showed significant herbicidal and antibacterial potencies [98]. Hydrogen peroxide determined substantial antioxidant, antibacterial and antifungal activity [2]. These *Paeonia* *emodi* Wall. ex Royle chemicals are antioxidative, antitumor, and antipathogenic, and they help to regulate the immune system, protect the cardiovascular system, and protect the central nervous system. [102]. The main phytoconstituents of *Paeonia* *emodi* Wall. ex Royle and their pharmacological properties are summarized in Table 2.

**Table 2:** Chemical constituents and their biological properties reported from *Paeonia* *emodi* Royle

Class of compounds	Chemical compound	Sources/part of plant	Biological effect	Refs.
Triterpenes	Emodinol Oleanolic acid β-amyrin Lupeol 24-methylenecycloartanol Cycloartenol Betulinic acid	Roots/seeds/flowers/tuber	Anticoagulant Cardioprotective ↓Lipoxygenase ↓β-glucuronidase Free-radicals Antibacterial Antiulcer	[96] [101] [56] [4] [35]
Monoterpenes	Lactiflorin, Paeonin A, B, C Wurdin Benzoyl wurdin Paeoniflorin Oxypaeoniflorin Paeoninol	Roots/tuber/seeds	↓Lipoxygenase Antioxidant ↓Free radicals Anticoagulant Cardioprotective Antibacterial	[35] [94] [96] [103] [98] [48] [104]
Phenolic acids	Paeonol Hydroxybenzoic acid Gallic acid Methyl gallate Ethyl gallate Methylgrevillate Benzoic acid 3-hydroxy benzoic acid 4-hydroxy benzoic acid Oligo stilbene Chrysophanic acid Syringic acid Ethyl gallate	Seeds/tuber/roots	Antioxidant ↓Free radicals ↓Lipoxygenase, ↓β-glucuronidase Anticoagulant Cardioprotective	[8] [98] [103]
Fatty acids	Octanoic acid Decanoic acid Lauric acid Myristic acid Palmitic acid Palmitoleic acid Stearic acid Oleic acid Linoleic acid Palmitoleic acid Myristoleic acid Myristic acids Butyrospermol	Roots	Antibacterial Antifungal Antioxidant	[100] [35] [104]
Steroids	Campesterol Sitosterol	Roots/tuber/seeds/petals	Antibacterial Antifungal Antioxidant	[2, 100] [35] [104]

### Pharmacological properties of *Paeonia emodi*

#### Antioxidant

Natural antioxidants are a group of bioactive compounds that can help support cell integrity by neutralising free radicals, unstable molecules that the human body produces [105]. These bioactive compounds are therefore essential and indispensable for the proper functioning of the body [106–109]. Consuming antioxidants can help the body get rid of extra reactive oxygen species and free radicals [110–114]. Many unprocessed extracts and pure chemicals derived from Paeonia species have been said to have free radical-scavenging action [6]. In comparison to the diabetic nephropathy control group, the *P.* *emodi* extract treatment enhanced the level of the antioxidant enzyme. Super oxide dismutase (SOD) levels significantly rose in the pancreas, liver, and kidney [4].

#### Anti-inflammatory 

Pain, redness and swelling are symptoms that betray inflammation and for treating these ailments conventional anti-inflammatory drugs are effective but have several side effects [115, 116]. To avoid the unwanted side effects of drugstore anti-inflammatories conventional anti-inflammatory drugs can be replaced with natural bioactive compounds [17, 19, 117, 118]. The literature is well aware of the significant anti-inflammatory properties of plant-based natural substances [91, 119–121]. It has been reported in earlier studies that the root extract of *P.* *emodi* containing poly- saccharides significantly reduced inflammation when tested in vivo on male albino rats [4]. During in vitro experiments, it is advised that *P.* *emodi* be examined to assess its anti-inflammatory potential. A focus of recent medical research has been the identification of innovative anti-inflammatory medicines derived from natural ingredients. The anti-inflammatory action of the genus *Paeonia* has received the most research attention [4].

#### Anticancer and cytotoxic properties

Cancer is a large group of diseases that vary in mode of onset, growth rate, diagnosis, detectability, invasive potential, metastasis, response to treatment and prognosis [122–126]. Numerous studies have also revealed that additional Paeonia constituents may be used to treat breast, lung, and liver cancers and leukaemia. The monoterpene 6′-*O*-galloylpaeoniflorin suppresses metastasis via the AMPK signalling system and has lethal effects on non-small-cell lung cancer cells [127]. There are records of the Paeonia genus being used to cure tumours, and contemporary pharmacological studies of plant extracts have partially verified its antitumor properties. Paeonia contains a variety of substances, primarily monoterpene glycosides and stilbenes, which have strong antitumor activity in vitro, further highlighting the potential benefits of Paeonia plants [6].

#### Anti-mutagenic

Cancer occurs as a result of somatic mutations in the cells that make up tissues [128–130]. Random mutations that constantly accumulate without having a negative impact on cell survival are passenger mutations and they do not cause clonal expansion of a malignant transformed cell, they do not promote tumour growth instead driver mutations cause oncogenesis [131, 132]*.* *P.* *emodi* plays a very important role in anti-mutagenic activity. The dried leaves extract of *P.* *emodi* is used in vitro to study the mutagenic effect. The extract demonstrated improved DNA protection and was able to reduce the oxidative stress brought on by the Fenton reaction [4]. However, it has been noted that more effort is required to complete the aforementioned task to achieve the proper mechanism [4].

#### Cardioprotective and antihyperlipidemic properties

The traditional uses of Paeonia species, which frequently entail promoting blood stasis and treating hematemesis, are connected to their cardiovascular preventive advantages. It is believed that the substances PF and paeonol may be useful in the treatment of cardiovascular diseases. The treatment of myocardial ischemia, myocardial infarction (MI), atherosclerosis, hypertension, inhibition of thrombosis, and improvement of myocardial remodelling are among the cardioprotective benefits of the genus Paeonia [4]. Hyperlipidemia is one of the primary causes of oxidative stress, a feeble antioxidant defence, diabetes, and nephropathy [133]. When fruit extracts were previously studied for the treatment of nephropathy, researchers found that this plant dramatically brought glucose levels back into the normal range [134].

#### Hepatoprotective

The liver is a crucial organ that manages several aspects of the digestive system and detoxifies xenobiotics produced by the body [135–137]. *P.* *emodi* extracts' hepatoprotective potential in methanol and ethanol has been studied [4].

#### Nephroprotective

Nephropathy is a frequent complication of life-threatening conditions like diabetes that can increase morbidity and mortality [138]. Researchers who previously investigated fruit extracts for the treatment of nephropathy discovered that this plant significantly reduced glucose levels to the normal range [139]. The root extract of *P.* *emodi* showed protective properties against diabetic nephropathy by improving blood glucose levels, associated diabetic neuropathy biomarkers, and advanced glycation end products in the kidney [4].

#### Antibacterial and antifungal properties

The bioactive compounds present in plants have been used for their beneficial therapeutic effects and in many preclinical pharmacological studies, their therapeutic potential for human health has been investigated. Up till now, the majority of microbes are investigated having resistance to antibiotics [140–145]. Therefore, to overcome this problem and obtain effective antimicrobial agents different plants are used by pharmaceutical companies to beat the issue of obstruction-breaking strains of microorganisms [19, 91, 146, 147]. Many scientists have paid attention to plant extracts used in herbal medicine because of the side effects and pathogenic microorganisms which have developed resistance to antibiotics [146, 148, 149]. From different parts of the world, scientists have investigated different medicinal plants with anti-microbial potential, which helped in processed food preservation, medications, natural remedies, and alternative drugs [91, 106, 117, 141, 143, 146]. Regarding the antibacterial activities of *Paeonia* *emodi* Wall. ex Royle a recent study has been carried out [39, 49]. 38 cases representing infection with nine different bacterial strains and treated with different doses of *Paeonia* *emodi* Wall. ex Royle extracts (n-hexane, chloroform, ethyl acetate, crude, ethanolic extract) have been analyzed. The different strains reported in the literature treated by *E.* *coli,* *P.* *aeruginosa,* *S.* *aureus,* *S.* *epidermidis,* *S.* *typhi,* *Proteus* *vulgaris,* *and* *K.* *pneumoniae* are all susceptible to *Paeonia* *emodi* Wall. ex Royle extracts in results, [49] reported maximum inhibition of *P.* *aeruginosa* and *S.* *aureus* using different extracts. In another extract maximum inhibition was observed in *P.* *aeruginosa* against (ethanolic extract), *S.* *aureus* against (ethanolic extract) and *P.* *vulgaris* against (ethanolic extract). The reason for maximum inhibition by ethanolic extract may be, that the solvent is more economical and its properties lie somewhat in between water and oil. Different fractions of *Paeonia* *emodi* Wall. ex Royle was utilized to decide the zones of inhibition of bacterial development on the agar plate [49]. It was reported by [49] *Paeonia* *emodi* Wall. ex Royle inhibited the growth of *K.* *pneumoniae*, *Salmonella* *typhi* and *P.* *aeruginosa*. *E.* *coli* showed different zone of inhibition in various extracts such as chloroform, ethyl, n-hexane and crude [49]. The different extracts were applied on Methicillin-resistant and *Staphylococcus* *aureus* and several zones of inhibition were obtained [49]. Usually, in the media MRSA was referred to as superbug and as a whole was multi-drug resistant [150]. *Paeonia* *emodi* Wall. ex Royle showed important antibacterial activity and was capable to be utilized in the treatment of irresistible ailments brought about by *E.* *coli,* *P.* *aeruginosa,* *S.* *aureus,* *K.* *pneumonia,* *S.* *epidermidis,* *S.* *typhi*, and MRSA microbes. For antifungal properties of *Paeonia* *emodi* Wall. ex Royle only a few fungal strains were tested because most of the researchers mainly focused on antibacterial activity. *Paeonia* *emodi* Wall. ex Royle demonstrated moderate antifungal movement. By negative control experiment invitro technique was applied and 400 µg of the extraction/ml of sabouraud dextrose agar was used. The ethanolic extract of *Paeonia* *emodi* Wall. ex Royle had the most substantial antifungal activity observed by the human pathogen *Pseudalleschena* *boydii* i-e 55.5% Animal pathogen *Microsporum* *canis* showed 55.1% antifungal activity by using ethanolic extract [24] while plant pathogen *Fsuarium* *solani* showed 50% antifungal activity. Further different models were used such as *Trichophyton* *schoenleinii,* *Candida* *albicans,* *Aspergilus* *niger,* *Trichophyton* *simii*, *and* *Macrophomina* *phaseolina* which showed significant antifungal activity. Thus, in nutshell, *Paeonia* *emodi* Wall. ex Royle displayed potential antibacterial activities against different bacterial strains (Table 3).

**Table 3:** Different *Paeonia* *emodi* Wall. ex Royle mediated extracts and their antibacterial potentials

Extract	Dose (mg/mL)	Zone of inhibition (mm)	Tested bacteria	Refs.
*n*-Hexane	10	14	*Escherichia* *coli*	[49]
Chloroform	10	14	*E.* *coli*	[49]
Ethyl acetate	10	12	*E.* *coli*	[49]
Crude	10	16	*E.* *coli*	[49]
*n*-Hexane	10	20	*Pseudomonas* *aeruginosa*	[49]
Chloroform	10	18	*P.* *aeruginosa*	[49]
Ethyl acetate	10	20	*P.* *aeruginosa*	[49]
Crude	10	16	*P.* *aeruginosa*	[49]
Ethanolic extracts	30	18 ± 0.7	*P.* *aeruginosa*	[39]
Ethanolic extracts	20	12 ± 0.9	*P.* *aeruginosa*	[39]
Ethanolic extracts	10	**-**	*P.* *aeruginosa*	[39]
*n*-Hexane	10	10	*Klebsiella* *pneumoniae*	[49]
Chloroform	10	16	*K.* *pneumoniae*	[49]
Ethyl acetate	10	20	*K.* *pneumoniae*	[49]
Crude	10	14	*K.* *pneumoniae*	[49]
*n*-Hexane	10	12	*Methicillin-resistant* *Staphylococcus* *aureus* *(MRSA)*	[49]
Chloroform	10	14	*MRSA*	[49]
Ethyl acetate	10	18	*MRSA*	[49]
Crude	10	14	*MRSA*	[49]
*n*-Hexane	10	12	*Staphylococcus* *aureus*	[49]
Chloroform	10	12	*S.* *aureus*	[49]
Ethyl acetate	10	18	*S.* *aureus*	[49]
Crude	10	14	*S.* *aureus*	[49]
Ethanolic extracts	30	16 ± 0.3	*S.* *aureus*	[39]
Ethanolic extracts	20	12 ± 0.1	*S.* *aureus*	[39]
Ethanolic extracts	10	–	*S.* *aureus*	[39]
n-Hexane	10	10	*Staphylococcus* *epidermidis*	[49]
Chloroform	10	18	*S.* *Epidermidis*	[49]
Ethyl acetate	10	16	*S.* *Epidermidis*	[49]
Crude	10	16	*S.* *Epidermidis*	[49]
*n*-Hexane	10	8	*Salmonella* *typhi*	[49]
Chloroform	10	18	*S.* *Typhi*	[49]
Ethyl acetate	10	20	*S.* *Typhi*	[49]
Crude	10	16	*S.* *Typhi*	[49]
Ethanolic extracts	30	–	*Proteus* *vulgaris*	[39]
Ethanolic extracts	20	–	*P.* *vulgaris*	[39]
Ethanolic extracts	10	19 ± 0.3	*P.* *vulgaris*	[39]
Crude	10	14	*K.* *pneumoniae*	[49]
Silver oxide	30	1.281	*B.* *subtilis*	[35]
Silver oxide	30	1.519	*S.* *aures*	
Silver oxide	30	1.370	*Escherichia* *coli*	
Silver oxide	30	1.661	*P.* *aeruginosa*	
Ethanolic extract	Not mention	19.27 ± 0.23	*S.* *marcescens*	[104]
Methanolic extract	Not mention	13.28 ± 0.12	*Actinobacteria*	[104]

#### Neuroprotective

The brain is one of the most important organs of the body, which allows the evolution of the human being and species [17, 111, 151–153]. Brain functions are influenced by poor nutrition, stress and lack of movement, but also by natural cellular oxidation processes [19, 106, 154–157]. In addition to improving lifestyle, the brain and memory can benefit from the stimulating input of nature due to plants with a beneficial effect on brain functions [158–160]. Recent research studied the effects of a *Paeonia* *emodi* Wall. ex Royle ethanol concentrate at doses ranging from 300 to 600 mg/kg BW on pentylenetetrazole-igniting, memory impairment, oxidative damage, and anxiety without engine debilitation. *Paeonia* *emodi* Wall. ex Royle has been demonstrated to be effective in the treatment of dropsy and worried concerns because of its cell reinforcement and radical rummaging features and activities [4].

#### Anticonvulsant, antiepileptic, antianxiety

One of the brain disorders, epilepsy is primarily brought on by psychological, physical, and social actions [112]. In a recent study, the researchers showed that the plant extract of *P.* *emodi* had anticonvulsant and antianxiety effects that were statistically significant [4].

### Enzyme inhibition and radical scavenging activities

#### Lipoxygenase inhibiting activity

Fruit extract of *Paeonia* *emodi* Wall. ex Royle was found to contain Paeoninol and Paeonin C, oligostilbene and monoterpene galactoside along with 4-hydroxybenzoic acid, gallic acid and methyl gallate. In the concentration-dependent method, these compounds exhibited powerful inhibitory potential against lipoxygenase enzyme showing IC_50_ values of 0.77 and 99.5 mM and with ABTS ± radical quenching activity with IC_50_ values of 147.5 and 498.2 µM [8].

#### β-glucuronidase inhibiting activity

*Paeonia* *emodi* Wall. ex Royle has been used to isolate emodinol from its chloroform soluble fractions. Spectral studies of arrangement 1beta, 3beta, 23-trihydroxyolean-12-en-28-oic acid have been allocated along with 2D NMR which has shown the significance of beta-glucuronidase inhibitory activities. The typical inhibitor, glucosaccharo-lactone, has an IC_50_ value of 1.88 mM, but emodinol derived from the roots of *Paeonia* *emodi* Wall. ex Royle has an IC_50_ value of 63 mM [43an]. Benzoic acid and 3-hydroxybenzoic acid have also been isolated from *Paeonia* *emodi* Wall. ex Royle [96]. From the above-ground parts of *Paeonia* *emodi* Wall. ex Royle extracts of ethanol were selected against Urease and alpha-chymotrypsin for enzyme inhibition and radical scavenging activity using the DPPH assay. Concentrates of unrefined against jack bean (74%) and *Bacillus* *pasteurii* (80%) urease indicated noteworthy catalyst restraint action and moderate movement (54%) against alpha-chymotrypsin while (83%) radical searching action was additionally gotten from the concentrate [48].

#### Urease activity

Mansoor and Taos (2005) evaluated the urease activity of *Paeonia* *emodi* in a 15-min experiment at 30 degrees Celsius. 5 µl of test compounds were incubated with 25ul of enzyme solution and 55ul of 100 mM urea buffers. Weather burn’s indophenol approach was used to test urease activity by generating ammonia. With the assistance of the SoftMax Pro software. The data was examined. Thiourea was an established urea inhibitor.

### Antispasmodic 

*Paeonia* *emodi* Wall. ex Royle was found to contain divisions of harsh concentrates from its aerial parts which indicated potential spasmolytic action. Effects of 5 mg/mL crude extract concentration were studied on jejunum rabbits inhibiting the spontaneous motility by 76%. Fractions containing ethyl acetate and chloroform showed excellent spasmolytic activities while the n-butanol fraction showed low inhibitory activities. A general spasmogenic action appeared from the portions of solvent water in the separated jejunum rabbit [48]. Tubers of *P.* *emodi* are used to study the uterotonic effect but no response was shown by the model organism [4].

### Other biological properties

The rough concentration of *Paeonia* *emodi* Wall. ex Royle was evaluated for phytotoxicity against *Lemna* *minor* *L*. Three flasks for 500, 50, and 5 g/ml were utilized with a stock arrangement of the concentrate (20 mg/ml). Each jar held 20 mL of medium. Paraquat was employed to suppress growth. Jars were hatched in the development area for seven days before the development guideline in rate was examined using a negative control. The IC_50_ value was determined. The PC program was operational 95 percent of the time, according to preliminary statistics [19]. The phytotoxicity of *Paeonia* *emodi* Wall. ex Royle was also tested against Lemna aequinoctialis. KOH pellets were mixed into the medium, which was composed of purified water with a pH of 5.5–6.5. The medium was autoclaved for 15 min at 121 °C. As a starting solution, ethanol extracts were utilized. In the experiment, nine fertilized flasks were used. Each flask contained a rosette of *Lemna* *aequinoctialis* fronds. In sterile conditions, the solvent was evaporated. All flasks were kept in a growth cabinet and plugged in for seven days. The number of fronds was counted on day seven [13].

The insecticidal efficacy of *Paeonia* *emodi* Wall. ex Royle separate was modulated by direct contact application using channel paper. 3 ml of the concentrate was put on channel papers. After drying, each channel paper was put in a separate petri dish with *Tribolium* *castaneum*, *Bruchus* *pisorum*, and *Rhyzopartha* *dominica*. Permethrin, a pesticide, was employed as a control. All were kept without food for 24 h before undergoing a mortality check [19]. 70% Ethanolic extracts prepared from the roots of *Paeonia* *emodi* Wall. ex Royle was used to examining its effects on the atria, trachea, and aorta of pigs and rats. All the techniques applied were in vivo. Similarly, airway relaxant effects were examined in the lungs of the mouse. Different results were obtained demonstrating, vasodilatory, antiplatelet, and tracheal and aviation route relaxant activities. Ethanolic extracts of *Paeonia* *emodi* Wall. ex Royle justified its importance as a medicinal herbal drug in treating various cardiovascular and respiratory ailments [161].

**Fig. 5:**
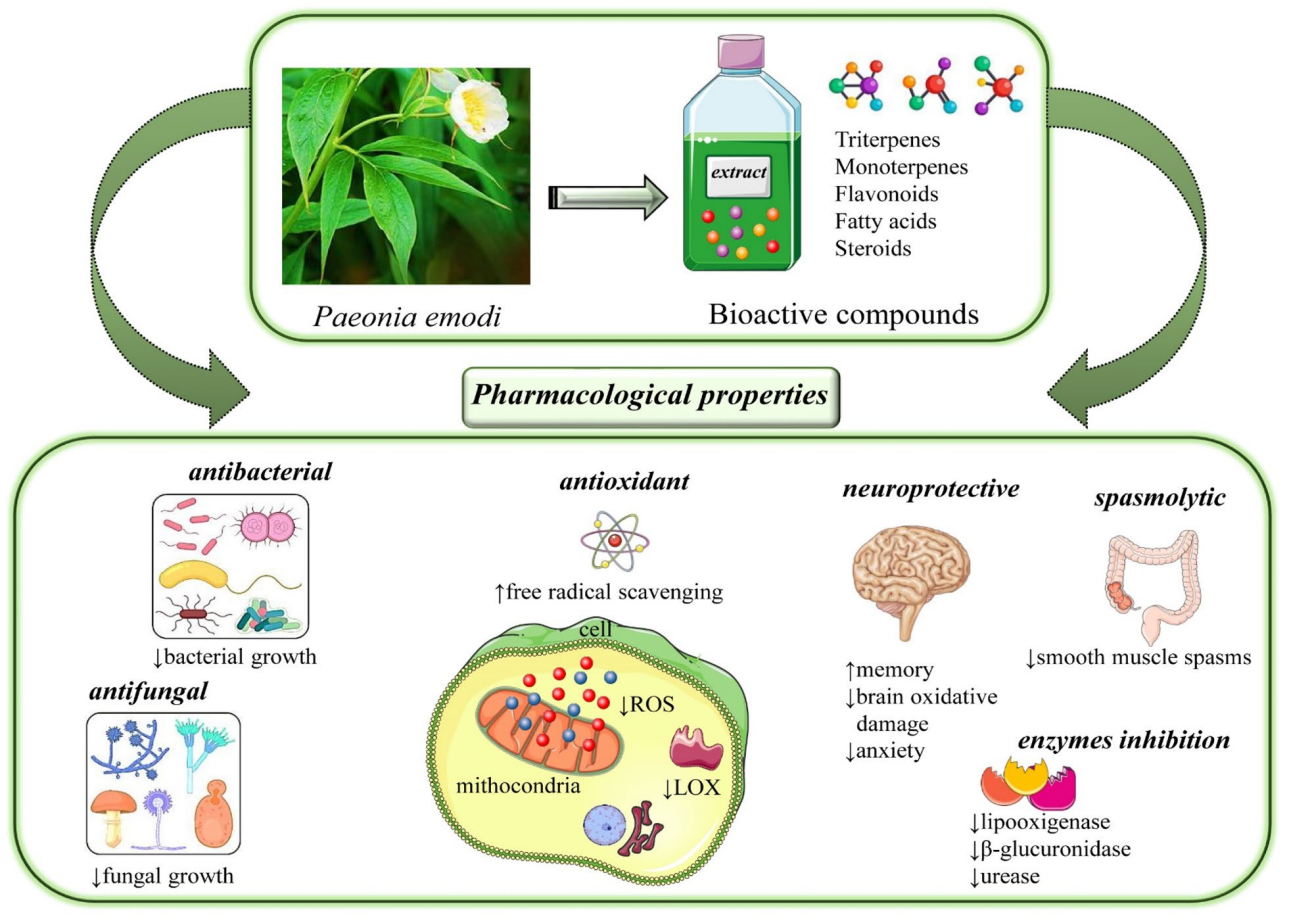
Summarized scheme with the main phytoconstituents of *Paeonia* *emodi* Wall. ex Royle and their most relevant pharmacological properties. Abbreviations and symbols: ↑ increase, ↓decrease, lipoxygenase (LOX), reactive oxygen species (ROS)

The most relevant data regarding pharmacological properties of *Paeonia* *emodi* Wall. ex Royle are summarized in Fig. 5 and Table 4.

**Table 4:** The main pharmacological properties of *P.emodi*

Pharmacological activity	Experimental study	Tested extract of *Paeonia* *emodi* Wall. ex Royle	IC_50_/dose	Main results	Refs.
Antibacterial	In vitro *Escherichia* *coli* *Proteus* *aeruginosa* *Staphylococcus* *aureus* *Staphylococcus* *epidermidis* *Salmonella* *typhi* *Proteus* *vulgaris* *Klebsiella* *pneumoniae*	*n*-hexane chloroform Ethyl acetate Ethanolic silveroxide	NA	↓ Bacterial growth	[49] [39] [35]
Antifungal	In vitro *Pseudalleschena* *boydii* *Trichophyton* *schoenleinii* *Candida* *albicans* *Aspergilus* *niger* *Trichophyton* *simii* *Macrophomina* *phaseolina*	Ethanolic Methanolic	IC_50_ = 400 µg/mL IC_50_ = 100–900 µg/mL	Moderate antifungal activity	[5, 24]
Neuroprotective	In vivo Mice PTZ epilepsy model	Roots ethanolic extract	Dose = 300–600 mg/kg/bw	↑Memory ↓Brain oxidative damage ↓Anxiety ↓Seizures	[4]
Enzymes inhibition	In vitro	Fruit ethanolic extract	IC_50_ = 147.5–498.2 µM	↓Lipoxygenase	[8]
		Roots ethanolic extract	IC_50_ = 1.88 mM	↓β-glucuronidase	[96]
Antispasmodic	In vivo Rabbits	Crude extract	Dose = 5 mg/mL/kg/bw	↓Smooth muscle spasms	[48]
Anti-inflammatory	In vivo Albino rats	Roots aqueous extract	Dose = 20 g/5 h	↓Pro-inflammatory factors	[4]
Hepatoprotective	In vivo Albino rats	NA/ethanol	Dose = NA/1 day	Hepatoprotective against ethanolic and methanolic toxicity	
Nephroprotective	In vivo Wistar rats	Roots/alcoholic/ Hydroalcholic extract	Dose = 100,200, 400 mg/kg/45 days	Protective effects against kidney damages	

Symbols and abbreviations: ↑ increase, ↓decrease, the half maximal inhibitory concentration (IC_50_), Body Weight. (bw), Pentylenetetrazole (PTZ), nonavailable (NA)

### Toxicology, side effects and safety data

For a decade, there has been a resurgence in interest in herbal remedies although the safety of herbal treatments has been repeatedly questioned [142]. There are currently misconceptions and prejudices about the safety of herbal medication [4]. According to the literature study, only a few research has been done on the toxicology of *P.* *emodi*. According to Zargar et al. [95], hydroalcoholic and aqueous plant extracts were deemed safe because they did not result in any mortality up to 2000 mg/Kg body weight. Herbal medicine side effects can have a variety of direct and indirect causes, which can be categorized. The inherent toxicity of several herbs, whether taken in overdose or at a regular therapeutic dosage, is a direct cause. Consumers may feel more secure if there is a regularity framework in place for herbal medications. The specification and regulation of herbal medications, however, differ significantly across nations. The World Health Organization (WHO) should suggest global unified planning, which includes global management standards and quality standards, radical sources of herbs, seeds and seedling breeding, planting, harvesting, and storage, rational procedures, manufacture, and quality standards to ensure the quality and safety of herbal medicines. Additionally, a system for ensuring the safety of herbal medicines should be built. This system should include prudent clinical practice and risk monitoring and play a bigger part in preserving human health [4].

### Limitations and clinical gaps

Although the bioactive chemical compounds found in the *Paeonia* *emodi* Wall. ex Royle species have demonstrated a variety of biological effects in preclinical pharmacological studies, this species cannot be used as the first-line treatment for many chronic conditions for the following reasons:

- There aren't enough clinical trials to back up these bioactive chemicals' toxicity, side effects, and therapeutic effects.
- The absence of translational pharmacological investigations to identify the optimal therapeutic dose and administration method for achieving the best possible therapeutic impact in humans.
- Insufficient experimental research on extracts to precisely describe the bioactive components after they have been purified. To ascertain the precise concentrations of bioactive chemicals that could be utilized in possible clinical trials, these extracts should be tested in several preclinical pharmacological experiments and chemically described.
- A lack of research on nanomedicines would boost the bioavailability and effectiveness of these bioactive substances by including them in nanocarriers in certain target tissues. The intention is to maximize bioavailability by including phytochemicals in carrier nanoparticles, both in terms of the target tissue/organ and in terms of the moment/time in which the included bioactive compound is released.

## Overall conclusions

*Paeonia* *emodi* Wall. ex Royle is an important restorative herb with a broad pharmacological spectrum. It is concluded from the present literature that *Paeonia* *emodi* Wall. ex Royle roots and rhizome are the most used part of the plant. There is an excessive possibility for the further screening of the plant against several disorders using both in vitro and in vivo animal models. A lot of work is needed to be done on the antimicrobial activity of this plant and more concern should be on data for the possible toxicity of the herb (toxicological studies, lethal dose, etc.). More phytochemical screening of this plant should be done to discover new bioactive phytochemical entities existing in the plant as this plant is the least misused species in the genus. In the future, we recommend different in vitro and in vivo biological studies using different animal models to further investigate its biopharmacological efficacies.

## Data Availability

The data supporting this review are from previously reported studies and datasets, which have been cited. The processed data are available from the corresponding author upon request.
